# Ultra-thin, zoom capable, flexible metalenses with high focusing efficiency and large numerical aperture

**DOI:** 10.1515/nanoph-2023-0561

**Published:** 2023-11-17

**Authors:** Yilin Shi, Hao Dai, Renjie Tang, Zequn Chen, Yalan Si, Hui Ma, Maoliang Wei, Ye Luo, Xingyi Li, Qing Zhao, Yuting Ye, Jialing Jian, Chunlei Sun, Kangjian Bao, Yaoguang Ma, Hongtao Lin, Lan Li

**Affiliations:** State Key Laboratory of Modern Optical Instrumentation, Key Laboratory of Micro-Nano Electronics and Smart System of Zhejiang Province College of Information Science and Electronic Engineering, Zhejiang University, Hangzhou 310027, China; Key Laboratory of 3D Micro/Nano Fabrication and Characterization of Zhejiang Province, School of Engineering, Westlake University, Hangzhou 310030, China; Institute of Advanced Technology, Westlake Institute for Advanced Study, Hangzhou 310024, China; State Key Laboratory for Extreme Photonics and Instrumentation, College of Optical Science and Engineering, Intelligent Optics and Photonics Research Center, Jiaxing Research Institute, ZJU–Hangzhou Global Scientific and Technological Innovation Center, International Research Center for Advanced Photonics, Zhejiang University, Hangzhou 310058, China; Najing Science and Technology, Hangzhou 310027, China

**Keywords:** dielectric metasurface, tunable metalens, zoom imaging

## Abstract

The ever-growing demand for miniaturized optical systems presents a significant challenge in revolutionizing their core element – the varifocal lens. Recent advancements in ultra-thin, tunable metasurface optics have introduced new approaches to achieving zoom imaging. However, current varifocal metalens have faced challenges such as low focusing efficiency, limited tunability, and complicated designs. Here, we employ the high-contrast transmit arrays (HCTA) structures to design and fabricate a polarization-independent, single-layer flexible metalens that operates at a wavelength of 940 nm. Using a uniform stretching system, we characterized its optical performance to achieve over 60 % focusing efficiency within a 0 %–25 % stretch range, while the focal length changes align with theoretical predictions. Furthermore, our research also successfully demonstrated the capacity of a metalens with a numerical aperture (NA) of 0.5 to efficiently adjust imaging magnification within a 2× range, achieving imaging results that approach the diffraction limit. This research offers promising prospects for the practical use of compact and miniaturized optoelectronic devices in fields like photography, mixed reality, microscopy, and biomedical imaging.

## 1 Introduction

Zoom lenses play a critical role in modern electronic devices, including mobile phones, smartwatches, and portable microscopes, as they enable optical zoom adjustment via the movement or rotation of one of the lenses. However, as the desired zoom ratio range expands, the moving distance of the optical elements also increases, resulting in a bulkier lens kit that is increasingly challenging to miniaturize. To address this issue, several solutions have been proposed to decrease the spatial size, including electro-optic lenses (EOL) [1, 2], fluid-based tunable lenses (FTL) [3–5], and tunable acoustic gradient index lenses (TAGIL) [6]. However, these mechanisms still heavily rely on traditional lenses, which are limited by spatial dimensions and lack the desired flatness and thinness required for further miniaturization of imaging systems. Consequently, their potential for advancing the development of compact imaging systems is constrained.

Recently, the metasurface with subwavelength thickness has emerged as a new type of flat optical platform [7] which can apply spatially varying transfer functions on incident wavefronts, including those of holograms [8–10], polarization elements [11–14], structure light projection [15, 16], steering gratings [17–19], and metalenses [20–26]. Metalenses [20–24, 26] composed of metasurfaces are thin and lightweight, showcasing higher focusing efficiency compared to conventional diffractive lenses (CDLs) and offering greater tunability [27]. In recent years, researchers have explored various approaches, including the utilization of liquid crystals [28], polarization rotation [29], the manipulation of MEMS-based or mechanically-operated dual-layer structures through horizontal [30, 31], axial [32], and rotational movements [33, 34], phase-change materials [35], and flexible substrates [36] to achieve zoom imaging functions as shown in Table 1.

**Table 1:** Varifocal metalenses with imaging performance.

Reference	Regulating mechanism	Structural complexity	Wavelength (μm)	NA	Lens size (mm)	Focusing efficiency	Zoom imaging ratio
Badloe et al. [28]	Liquid crystals	1 layer	0.633	0.19, 0.1	1.5	0.435, 0.44	Dispersed
Aiello et al. [29]	Polarization rotation	1 layer	0.483–0.620	0.04–0.09	0.04	0.05–0.5	Dispersed
Colburn et al. [30]	Lateral displacement	2 layers	1.55, 0.633	–	10	0.57, 0.15	4 × (Continuous)
Han et al. [31]	Lateral displacement	2 layers	1.55	0.42	0.2	–	Dispersed
Arbabi et al. [32]	MEMS	2, 3 layers	0.915	0.014	0.5	>0.4	10.3 × −11.3 × (Continuous)
Wei et al. [33]	Mutual rotation	2 layers	1.55	0.164	1	0.54	18 × (continuous)
Luo Y et al. [34]	Mutual rotation	2 layers	0.491, 0.532, 0.633	0.079	1.6	0.35	Continuous
Shalaginov et al. [35]	Phase change material	1 layer	5.2	0.35, 0.45	1.5	0.237, 0.216	Dispersed
Wei et al. [36]	Stretching	1 layer	0.45, 0.55, 0.65	0.2	0.15	0.3	1.08 × 1.1 × (Dispersed)
This work	Stretching	1 layer	0.94	0.5	1,0.2	0.6–0.75	2 × (Continuous)

Among these techniques mentioned, the focusing efficiency of liquid crystal-based zoom metalenses and polarization-controlled [29] zoom metalenses is limited due to their dependence on the polarization direction of the incident light. MEMS-based or mechanically-operated Alvarez metalenses [30, 31] and moiré lenses [33, 34] offer the potential for achieving high zoom ratios through rotational or axial movement. However, their reliance on a dual-layer lens structure introduces challenges in system alignment and transmittance, resulting in relatively low focusing efficiency. Phase-change materials [35] offer non-volatile properties and hold promise for effective and continuous control, but achieving uniform and stable voltage tuning for large-sized metalenses poses challenges. Therefore, the current tunable metalenses based on phase-change materials mainly rely on thermal tuning [37]. However, the thermal tuning speed is insufficient to meet annealing requirements, resulting in one-directional control of phase-change materials, and limiting their ability to achieve repeated focal length adjustments. The zoom metalens based on a flexible substrate does not impose additional requirements on the polarization of incident light. Moreover, zoom imaging only requires a single-layer structure, allowing higher transmission efficiency. Additionally, flexible substrates can achieve a wide range of stable repeated focusing [38]. However, current demonstrations primarily focus on low numerical aperture (NA) and polarization-dependent flexible metalenses [38, 39], which may limit their use in higher-resolution imaging and broader application scenarios. Furthermore, there have been no theoretical analyses or experimental demonstrations showing the capability of flexible metalenses for zoom imaging.

In this study, to ensure uniform and stable stretching while maintaining high transmittance, we employed a polarization-insensitive metalens based on the high-contrast transmit arrays (HCTA) structure. We investigated the uniform stretching characteristics of metalens with 200 μm dimensions and an NA of 0.15. The metalens consistently maintained high focusing efficiency (>60 %) across different stretching levels (0 %–25 %), demonstrating focal length variations consistent with theoretical analysis. Importantly, we established the relationship between zoom magnification and strain. Using flexible metalens with an NA of 0.5, we successfully achieved high-resolution continuous imaging results approaching the diffraction limit within a 2× magnification range.

## 2 Principle

The flexible metalens design utilizes transmissive phase-type metasurface, enabling stable phase distribution and high transmittance during stretching. In transmission phase type metasurface, the phase change Δ*φ* primarily depends on the effective index difference Δ*n*_eff_ and thickness *t* of the transmission medium layer, which is mathematically expressed in Eq. (1).

(1) $$\begin{array}{c} \Delta \varphi =\frac{2\pi }{\lambda }\Delta n_{\text{eff}}t \end{array}$$

Based on the effective refractive index theory, transmission phase type metasurfaces offer a stable output phase by varying the effective refractive index Δ*n*_eff_, which can range from Δ*n*_eff_ ≈ 1 to Δ*n*_eff_ ≈ *n*_meta_, depending on light propagation in either the background medium or the metasurface [40]. This property makes waveguide transmission-type metasurfaces more suitable for flexible metalens designs due to their capacity to confine energy within the metasurface, leading to consistent and reliable phase output. Specifically, we use a transmission phase type metasurface composed of HCTAs [41–43] to achieve high transmission efficiency and stable phases of metalens. HCTAs were originally designed for highly efficient lenses through the materials with high refractive index contrast, such as amorphous silicon (α-Si) and SiO_2_, which efficiently localize energy near high refractive index materials, minimizing the influence of duty cycle on metasurface phase and transmission. This is demonstrated by the similar transmission and phase properties of HCTAs in both hexagonal and square lattice configurations [41].

According to Eq. (2), we designed a flexible metalens with a focal length of *f* at the wavelength of *λ*. We define the stretch ratio *ε* as the ratio of the deformed size of the lens after stretching to the original size of the lens before stretching. As the stretch ratio *ε* increases, the period *p* of the metasurface units gradually increase, resulting *r* change to *r*′ while maintaining a nearly constant output phase. This produces a uniformly varying hyperbolic phase profile $\varphi \left(r,\lambda \right)$ , resulting in a uniform change in focal length *f*′. As shown in Supporting Information part 1, when the object distance *u* remains constant, the magnification *α* of the image varies according to the Gaussian imaging formula [44]. The relationship between the image magnification *α* and the stretch ratio *ε* can be expressed by the following Eq. (3) (see Figure S1 in Supporting Information):

(2) $$\begin{array}{c} \varphi \left(r,\lambda \right)=-\frac{2\pi }{\lambda }\left(\sqrt{r^{2}+f^{2}}-f\right) \end{array}$$

(3) $$\begin{array}{c} \alpha =\frac{{\left(1+\varepsilon \right)}^{2}f}{u+{\left(1+\varepsilon \right)}^{2}f} \end{array}$$

Eq. (3) relates the magnification *α* of a uniformly stretched flexible metalens to the applied stretch ratio *ε*, object distance *u*, and initial focal length *f*. Specifically, when the initial object distance *u* and initial focal length *f* are fixed, the magnification *α* increases with the stretch ratio *ε*. Moreover, larger initial focal lengths *f* and smaller object distances *u* lead to a more significant effect of stretching on the magnification *α*.

## 3 Simulation

**Figure 1:**
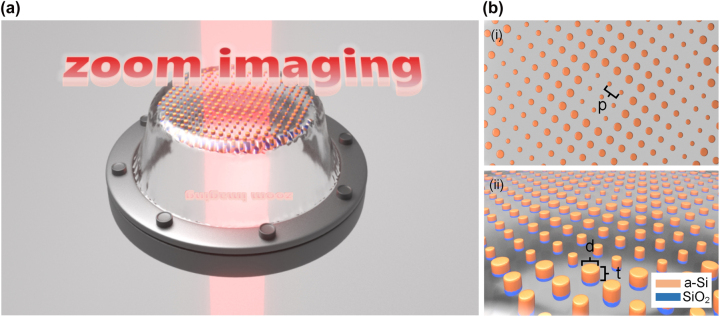
Schematic diagram of the flexible metalens during stretching, with descriptions for relevant structural parameters. (a) Schematic diagram illustrating the imaging of the metalens upon stretched PDMS. (b) Detailed schematics of the metasurface in stretched PDMS: (i) top view of the structural details of the stretched metalens, where the nano cylinders are arranged in a square lattice with a period denoted by ***p***. (ii) side view of the structural details of the stretched metalens, which is composed of amorphous silicon and silicon dioxide nanocylinders with the diameter and embedding depth denoted by ***d*** and ***t***, respectively.

The HCTA structure, illustrated in Figure 1(a and b), comprises α-Si cylinders with SiO_2_ masks embedded in a PDMS film. The refractive indices of α-Si, SiO_2_, and PDMS are 3.14, 1.45, and 1.46, respectively. To ensure the equation *φ* = 2π is obtained over a subwavelength propagation length, the thickness of the metasurface units must meet certain initial conditions [40].

(4) $$\begin{array}{c} \varphi =\frac{\Delta n_{\text{eff}}*t}{\lambda *2\pi } \end{array}$$

**Figure 2:**
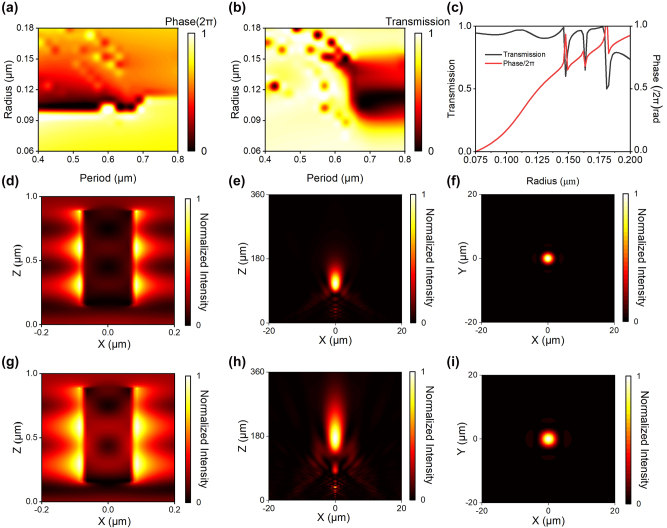
Simulation results of the flexible metasurface units and flexible metalens. (a) Phase distribution of cylindrical units with different periods and radius. (b) Transmission distribution of cylindrical units with different periods and radius. (c) Final selected units’ transmission and phase. (d) Electric field distribution of the unit with a period of 0.5 µm and a diameter of 0.12 µm before stretching. (e) Simulated optical intensity profiles of the unstrained (***ε*** = 0 %) metalens in the axial plane. (f) Simulated optical intensity profiles of the unstrained (***ε*** = 0 %) metalens in the focal plane. (g) Electric field distribution of the unit with a period of 0.54 µm and a diameter of 0.12 µm after stretching. (h) Simulated optical intensity profiles of the strained (***ε*** = 25 %) metalens in the axial plane. (i) Simulated optical intensity profiles of the strained (***ε*** = 25 %) metalens in the focal plane.

By substituting the refractive indices of the α-Si, SiO_2_, and PDMS into Eq. (4), we have identified the depth range of nanocylinders that achieves a 2π phase change. We then conducted numerical simulations using the finite-difference time-domain (FDTD) method to obtain the transmission and phase and generated metasurface unit library through a scanning process, as depicted in Figure 2(a) and (b). Specifically, to examine the transmission and phase variation of the units in different stretching states, we examined the transmission and phase of height *t* = 740 nm nanocylinders with diameters *d* varying from 120 to 360 nm in a period *p* ranging from 400 to 800 nm under the incidence of a normally incident plane wave at a wavelength of 940 nm.

Figure 2(a) and (b) demonstrate that for nanocylinders with a radius less than 0.1 µm, the transmission and phase remain relatively constant with the period, while for larger cylinder radii, slight fluctuations occur. To quantify this phenomenon, we analyzed the phase and transmission variation curves of a unit with a 0.12 µm diameter, which exhibited phase and transmission variations of 0.2 rad and 0.01 as the period *p* changed from 400 to 550 nm, respectively (see Figure S2 in Supporting Information). Due to the minuscule magnitude of these fluctuations, they can be considered negligible. Finally, a series of periodic HCTAs with a lattice constant *p* = 500 nm and height *t* = 740 nm was identified, comprising different scatterers that produced transmission amplitudes greater than 80 % and phases covering the entire 0 to 2 pi range, as demonstrated in Figure 2(c).

To understand the effect of maintaining a stable phase with high transmission, we analyzed the electromagnetic distribution of the designed unit with a diameter *d* of 120 nm under different periods *p* between 500 nm (*ε* = 0 %) and 540 nm (*ε* = 8 %), as illustrated in Figure 2(d) and (g), respectively. In Figure 2(d), the light is concentrated within the posts, which exhibit characteristics of weakly coupled, low-quality factor resonators. Even with further expansion of the period, the energy remains localized within the metasurface unit, indicating minimal changes in the electromagnetic field, as shown in Figure 2(g). This ability to maintain a stable phase distribution is essential for the functionality of the flexible metalens, as it ensures accurate and reliable focusing capabilities.

Due to limited computational resources, we simulated a flexible metalens with a diameter of 40 μm and a focal distance of 110 μm using the same numerical aperture used in the following experiment. The longitudinal light intensity profiles plotted in Figure 2(e) indicate that the unstressed metalens (*ε* = 0 %) has a focal length of approximately 110 μm, which matches the design focal length. Figure 2(f) presents the simulated intensity profile at the focal plane of the unstressed metalens, which displays a full width at half maximum (FWHM) of 2.68 μm. This value meets the criterion for diffraction-limited focusing (FWHM ≈ 0.51*λ*/NA), indicating that the metalens we have fabricated possesses imaging resolution capabilities approaching the diffraction limit. We define focusing efficiency as the ratio of the incident light that passes through a circular aperture in the plane of focus with a radius equal to three times the FWHM spot size. The simulation results shown in Figure 2(f) indicate that the flexible metalens has 72 % focusing efficiency on an unstressed state. Furthermore, as shown in Figure 2(e)–(h). The focal length of the metalens has changed from 110 μm (*ε* = 0 %) to 171 μm (*ε* = 25 %), and this result is consistent with the theoretical analysis (≈171.88 μm) (see equation (8) in Supporting Information). The decrease in focusing efficiency is due to the further expansion of the unit period (with a radius >0.1 μm) as it is stretched, causing phase variations in some units to deviate from the theoretical design. This leads to the appearance of secondary focal points, as shown in Figure 2(h).

## 4 Sample fabrication

**Figure 3:**
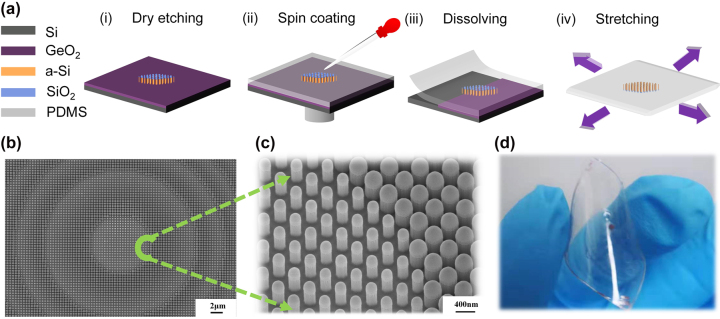
Major steps and key results in fabricating flexible metalens. (a) Major steps in fabricating flexible metalens: (i) Dry etching (ii) spin coating (iii) dissolving (iv) stretching. (b) Scanning electronmicrograph of the nanocylinders after dry etching, taken at an angle of 0°. (c) Scanning electron micrograph of the nanocylinders after dry etching, taken at a tilt angle of 30°. (d) Schematic diagram of the fabricated flexible metalens showing excellent flexibility.

Figure 3(a) (i–iv) illustrates the fabrication process for flexible metalenses. Initially, a germanium sacrificial layer with a thickness of around 800 nm is deposited onto a silicon wafer using magnetron sputtering. Next, the sample is annealed in a furnace under O_2_ atmosphere, which converts the germanium layer into GeO_2_. GeO_2_ is an ideal sacrificial layer due to its high solubility in water [45], enabling a simplified sample preparation process without harsh solvents like acids or hydrogen peroxide that may damage the metasurface units. Then, a 740 nm thick a-Si layer and a 350 nm thick SiO_2_ layer were deposited on the sacrificial layer through plasma-enhanced chemical vapor deposition (PECVD, Samco). After defining structures using e-beam lithography (EBL, Raith Voyager), the resist pattern was developed with maD-525 resist developer, followed by dry etching in an inductively coupled plasma (ICP, Samco) equipment (Figure 3(a), i). To ensure that the structure size is consistent with the design, we inspected the size of the metasurface units under a scanning electron microscope (SEM, Hitachi) system. Figure 3(b) presents the etching results of the sample, demonstrating the consistency in structure between the experiment and simulation. After etching, the sample was spin-coated with PDMS (10:1 mixing ratio of Sylgard 184 base and curing agent) to fill the gaps between the nano-posts (Figure 3(a), ii) and then degassed and cured in a vacuum oven. However, A single-layer PDMS coating is inadequate for achieving optimal stretching and conformal properties. While spin-coating a thicker layer may seem like a solution, it can result in excessive tension and fail to fill the nanopillar voids. In this study, we coat another 50 μm PDMS film on top of the initial 50 μm PDMS layer to enhance the stretchability of the flexible metalens.

The entire sample was degassed and cured in the vacuum oven. Subsequently, the sample was immersed in deionized water (DI water) to dissolve the sacrificial layer (Figure 3(a), iii). After 2 h, the sample with flexible metalens detached from the handling rigid substrate and released to be stretchable (Figure 3(a), iv).

## 5 Measurement procedures and results

**Figure 4:**
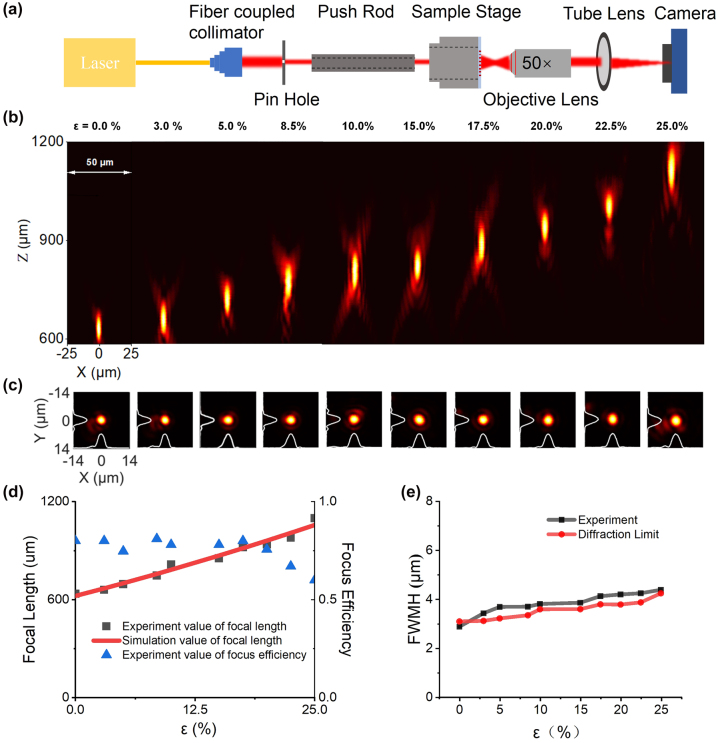
Test setup, simulation, and experimental results for characterizing the optical performance of the flexible metalens at different stretch ratios *ε*. (a) Optical path for characterizing the optical performance of the flexible metalens. (b) Measured optical intensity of flexible metalens in the axial plane at stretch ratios *ε* ranging from 0 % to 25 %, respectively. (c) Measured optical intensity of flexible metalens in the focal plane at stretch ratios *ε* ranging from 0 % to 25 % (0 %, 3 %, 5 %, 8.5 %, 10 %, 15 %, 17.5 %, 20 %, 22.5 %, 25 %, respectively). (d) Measured and simulated focal length as a function of stretch ratio ***ɛ***, as well as measured focusing efficiency. (e) Measured and diffraction limited FWHM spot size in the focal plane as a function of different stretch ratio ***ɛ***.

To achieve uniform stretching of the flexible metalens, we have designed and manufactured a uniform stretching device (more details in Supplementary Figure S3(a)). Firstly, to conduct the 0 % stretching test, the sample was mounted onto a glass slide and then subjected to varying stretch ratios *ε* for testing. The experimental setup utilized for assessing the performance of the flexible metalens is illustrated in Figure 4(a). In Figure 4(a), a single-mode fiber laser with a wavelength of 940 nm was used as the light source, which passed through a collimating lens to achieve collimation. Then, a tunable pinhole aperture that matches the size of the metalens was introduced in front of it to reduce the background noise. An objective lens and a tube lens with a focal distance of 125 mm were used to image intensity at different positions to a charge-coupled device (CCD, Sony).

The focal length is measured by using an electrically controlled translation stage to determine the distance from the focal point to the metalens. The power tests, including transmission and focusing efficiency, are carried out in a dark environment to prevent any interference from ambient air light that could potentially impact the accuracy of the results. The transmission is calculated by measuring the optical power passing through the lens and dividing it by the total power incident on the lens. The focusing efficiency is calculated by measuring the optical power near the focused spot, approximately three times the FWHM diameter and dividing it by the total power before the lens.

Using the setup illustrated in Figure 4(a) and the measurement procedure above, we determine the focal length, transmittance, focusing efficiency, and other key performance indicators of the flexible metalens under varying stretch ratios (*ɛ* = 0 % to *ɛ* = 25 %). Figure 4(b) demonstrates that the axial energy distribution of the flexible metalens changes with varying stretch ratios (*ɛ* = 0 % to *ɛ* = 25 %), resulting in a shift of the focal length from 636 μm to 1098 μm. Moreover, we have used a numerical fitting approach to derive Eq. (5) from the measured data shown in Figure 4(d), which relates the measured focal length *f*′ to the stretch ratio *ɛ* and the initial focal length *f*.

(5) $$\begin{array}{c} f^{\prime }\approx {\left(1+1.056\varepsilon \right)}^{2}f \end{array}$$

And it shows a good agreement with the theoretical results for focal length *f*′ with stretch ratio *ε* shown in Supplementary Eq. (8). Figure 4(c) indicates the energy distribution of the metalens on the focal plane under varying stretch ratios (*ε* = 0 % to *ε* = 25 %), revealing an increase in the FWHM of the focal spot during stretching while maintaining uniformity across various stretch ratios (*ε* = 0 % to *ε* = 25 %). The measured FWHM under various stretch ratios was compared with the theoretical values, as depicted in Figure 4(e), which also showed excellent consistency. The results for the metalens’ focusing efficiency at different stretch ratios *ε* are presented in Figure 4(d). As the stretch ratio *ε* increases, the focusing efficiency of the metalens decreases slightly from 83 % for no strain, with a more pronounced decrease starting at a stretch ratio of 15 %. This experimental result is consistent with the simulation results presented in Figure 2(b), which indicate that increasing the period from 500 nm to 625 nm (*ε* = 0 % to *ε* = 25 %) has little effect on transmittance for metasurface units with radius <0.15 μm. However, for a radius ranging from 0.15 μm to 0.18 μm, transmittance fluctuates significantly when the period is increased to 575 nm (*ε* = 15 %). This may lead to a slightly reduced focus efficiency of the fabricated flexible metalens after being stretched to 115 %. Nonetheless, the flexible metalens maintains a focusing efficiency of over 60 % within the stretching ratio between 0 % and 25 %.

## 6 Imaging results

**Figure 5:**
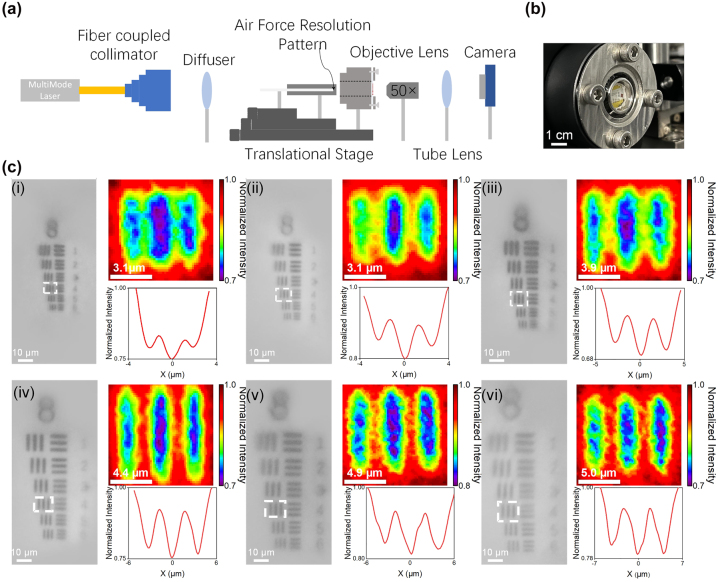
Test setup and experimental results for characterizing the imaging performance of the flexible metalens at different stretch ratios. (a) Optical path for characterizing the imaging performance of the flexible metalens; (b) schematic diagram of the uniform stretching device for imaging; (c) imaging results of the flexible metalens under different magnifications: 1×, 1.2×, 1.4×, 1.6×, 1.8×, and 2× (with the unstretched metalens image as 1×) shown in (i)–(vi). The scale bar for these images is 10 μm. A magnified photograph with the selected set of stripes and the corresponding average intensity analysis results are provided on the right side of each image.

To demonstrate the flexible metalens’ high-resolution imaging capabilities at different stretch ratios, we used a metalens with an NA of 0.5. Figure 5(a) illustrates the optical setup used to evaluate the imaging performance of the metalens under various stretch ratios. Firstly, as depicted in Figure 5(b), the metalens is secured on the sample stage of uniform stretching imaging characterization device by a metal sheet, and a quartz wafer with a resolution test chart deposited by metal deposition on its surface is attached to the front end of a glass rod to approximate the metalens. Then, a multimode fiber laser with a wavelength of 940 nm was used as the light source, and after passing through a diffuser, it illuminated a glass rod and projected resolution test charts. To maintain a consistent object distance while imaging at different stretch ratios, we utilized a translational stage to position both the glass and push rod. By incrementally advancing the push rod, we stretched the sample while simultaneously making slight adjustments to the position of the microscope objective after each stretch. This ensured proper alignment of the focal plane of the objective with the metalens’ image plane. The image procured by the metalens was then collected through a tube lens and projected onto a CCD camera, as shown in Figure 5(c) (i–vi). The imaging results of the unstretched metalens served as a fundamental reference for comparison. With increasing stretch ratios of the flexible metalens, it attained precise and continuous control of image magnification, with relative magnification ratios of 1.0×, 1.2×, 1.4×, 1.6×, 1.8×, and 2× (more details in Supplementary Figure S4). The theoretical resolution limits corresponding to relative magnifications of 1.2×, 1.4×, 1.6×, 1.8×, and 2× are 0.9579 μm, 1.01 μm, 1.06 μm, 1.109 μm, 1.14 μm, and 1.16 μm, respectively. We evaluated the quality of the imaging by measuring the average intensity of the fourth set of vertical stripes at various magnification levels, as demonstrated in the interpolation on the right-hand side of Figure 5(c) (i–vi). The research findings show that flexible metalenses can produce images with a resolution close to the diffraction limit at different stretch ratios, highlighting the potential of metalenses to achieve superior imaging quality with continuous zooming.

We also investigated the influence of repeatable stretching on the focusing and imaging performance of the flexible metalens with an NA of 0.5. Specifically, the flexible metalens was stretched over 2000 cycles from 0 % to 25 % and released from 25 % to 0 %. After around 2000 stretching and releasing cycles, the optical intensity of flexible metalens in the focal plane remained unchanged, and there was no observable impact on the imaging quality when imaging the same object (more details in Supplementary Figure S5). This indicates that the flexible metalens exhibits strong stretching reliability.

## 7 Conclusions

In summary, we have proposed a solution for miniaturizing zoom lenses by introducing single layer metalens based on a flexible substrate. Firstly, we presented the operational principle of the zoom lens that relies on a flexible metalens, establishing the relationship between magnification and stretch ratio. Subsequently, we conducted a comprehensive analysis of the metalens at different stretch ratios using a uniform stretching device. The results showed excellent performance of our fabricated flexible metalens at different stretch ratios, which closely approximates theoretical predictions. Moreover, we have successfully showcased the experimental stretch imaging control capability of our flexible metalens, providing evidence of a significant 2× range of free magnification control. In the future, building upon our developed process for flexible metalens fabrication and testing platform, we will leverage the conformal and stretchable properties of flexible substrates to explore novel applications of flexible metalenses such as large-field-of-view [46–48] and light-field imaging [49, 50].

## Supplementary Material

Supplementary Material is available online. It includes further details on the principle of zooming imaging based on flexible metalens and details of the phase and transmission changes caused by stretching in the most affected metasurface unit with a radius of 0.12 μm.

## Supplementary Material

Supplementary Material Details
