# Supplementary material for: Ultra-thin, zoom capable, flexible metalenses with high focusing efficiency and large numerical aperture
Source: Nanophotonics. 2023 Nov 17;13(8):1339–49. doi: 10.1515/nanoph-2023-0561 (PMC11636449; doi:10.1515/nanoph-2023-0561)
Supplement: Supplementary file 1 — Supplementary Material Details [file j_nanoph-2023-0561_suppl_001.docx]

Supplementary Information

Ultra-thin, Zoom Capable, Flexible Metalenses with High Focusing Efficiency and Large Numerical Aperture

Yilin Shi^1,2,3^, Hao Dai^1^, Renjie Tang^2,3^, Zequn Chen^2,3^, Yalan Si^1^, Hui Ma^1^, Maoliang Wei^1^, Ye Luo^2,3^, Xingyi Li^4^, Qing Zhao^5^, Yuting Ye^2,3^, Jialing Jian^2,3^, Chunlei Sun^2,3^, Kangjian Bao^2,3^, Yaoguang Ma^4*^, Hongtao Lin^1*^, and Lan Li^2,3*^

^1^State Key Laboratory of Modern Optical Instrumentation, Key Laboratory of Micro-Nano Electronics and Smart System of Zhejiang Province College of Information Science and Electronic Engineering, Zhejiang University, Hangzhou 310027, China

^2^Key Laboratory of 3D Micro/Nano Fabrication and Characterization of Zhejiang Province, School of Engineering, Westlake University, Hangzhou 310030, China

^3^Institute of Advanced Technology, Westlake Institute for Advanced Study, Hangzhou 310024, China

^4^ State Key Laboratory for Extreme Photonics and Instrumentation, College of Optical Science and Engineering, Intelligent Optics and Photonics Research Center, Jiaxing Research Institute, ZJU–Hangzhou Global Scientific and Technological Innovation Center, International Research Center for Advanced Photonics, Zhejiang University, Hangzhou 310058, China.

^5^Najing Science and Technology, Hangzhou 310027, China

*Correspondence and requests for materials should be addressed to Lan Li. (email：[lilan@westlake.edu.cn](mailto:lilan@westlake.edu.cn)), Hongtao Lin. (email:[hometown@zju.edu.cn](mailto:hometown@zju.edu.cn)) and Yaoguang Ma. (email:mayaoguang@zju.edu.cn).

1. **The illustration and principle of zooming imaging based on flexible metalens**


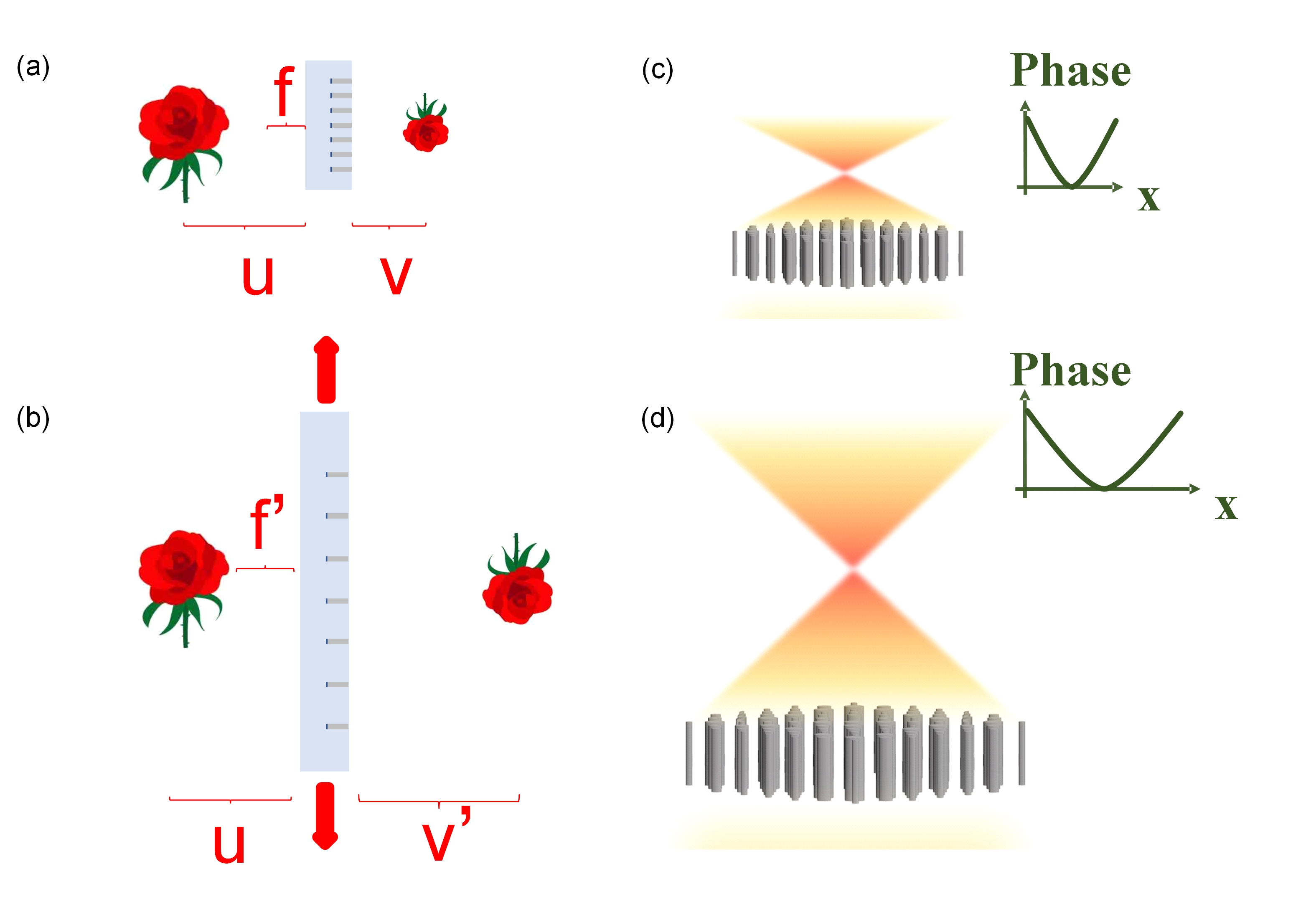


Supplementary Figure S1. Illustration and principle of zooming imaging based on a flexible metalens. (a) Imaging illustration before stretching the flexible metalens. (b) Imaging illustration after stretching the flexible metalens. The object distance is represented as $u$, image distances before and after stretching are $v$ and $v'$, and focal lengths before and after stretching are $f$ and $f'$. (c) Unstretched schematic diagram of the flexible metalens and its phase distribution on the x-axis. (d) Stretched schematic diagram of the flexible metalens and its phase distribution on the x-axis.

According to the Gaussian imaging Eq. 1, a change in the imaging system's focal length $f$ while maintaining a constant object distance $u$ results in a corresponding alteration in the image distance $v$. Hence, the axial magnification $\alpha$, expressed as the ratio of image distance $v$ to object distance $u$ shown as Eq. 2, can be tuned by adjusting the imaging system's focal length $f$, shown by Eq. 3.

|  | $\frac{1}{f}=\frac{1}{v}+\frac{1}{u}$ | (1) |
| --- | --- | --- |
|  | $\alpha=\frac{v}{u}$ | (2) |
|  | $\alpha=\frac{f}{u+f}$ | (3) |

The schematic diagram in Figures S1(a) and (b) illustrates the zooming imaging capability of a flexible metalens. Specifically, stretching the flexible metalens increases its focal length, changing the magnification of the image while keeping the object distance constant. Figure S1(c) and (d) show the paraxial phase profile of a flexible metalens in two states: unstretched and stretched, respectively. The relationship between the phase distribution $\varphi$ and focal length $f$ in the unstretched state is given by Eq. 4.

|  | $\varphi\left( r,\lambda\right)=\frac{2\Pi}{\lambda}(\sqrt{r^{2}+f^{2}}-f)$ | (4) |
| --- | --- | --- |

If the strain across a flexible metalens is uniform, a deformation of $\varepsilon$ causes a change in distance between the unit and center from $r$ to $r'(r'=r(1+\varepsilon))$, resulting in a new phase distribution $\varphi\left( r',\lambda\right)$ and a corresponding focal length of $f'$ , as shown in Figure S1(d) and Eq. 5.

|  | $\varphi\left( r',\lambda\right)=-\frac{2\Pi}{\lambda}(\sqrt{{r'}^{2}+{f^{'}}^{2}}-f')$ | (5) |
| --- | --- | --- |

Assuming that the local phase transformation is independent of the substrate deformation, which implies that the original phase at $r$ is equal to the phase at $r^{'}$ , therefore the relationship between $f^{'}$and $f$ can be deduced from the following Eq. 8. By substituting Eq. 8 into Eq. 3, we can derive the relationship between the axial magnification $\alpha'$ after stretching, stretch ratio $\varepsilon$, initial focal length $f$, and object distance $u$ shown as Eq. 9. We define the relative magnification ratio $Z$ as the ratio between the magnification factors $\alpha'$ of the lens after stretching and the magnification factor $\alpha$ of the lens before stretching, as shown in Eq. 10.

|  | $\varphi\left( r,\lambda\right)=\varphi\left( r^{'},\lambda\right)$ | (6) |
| --- | --- | --- |
|  | $-\frac{2\Pi}{\lambda}\left( \sqrt{r^{2}+f^{2}}-f \right)=-\frac{2\Pi}{\lambda}(\sqrt{{r^{'}}^{2}+{f^{'}}^{2}}-f') ( r’=r\left( 1+\varepsilon\right) )$ | (7) |
|  | $f^{'}\approx{(1+\varepsilon)}^{2}f$ | (8) |
|  | $\alpha'=\frac{{(1+\varepsilon)}^{2}f}{u+{(1+\varepsilon)}^{2}f}$ | (9) |
|  | $Z=\frac{\alpha'}{\alpha}=\frac{\left( 1+\varepsilon1 \right)^{2}(u+\left( 1+\varepsilon2 \right)^{2}f)}{(u+\left( 1+\varepsilon1 \right)^{2}f){(\left( 1+\varepsilon2 \right)}^{2})}$ | (10) |

1. The phase and transmission changes caused by stretching in the metasurface unit with a diameter of 0.12 microns

**
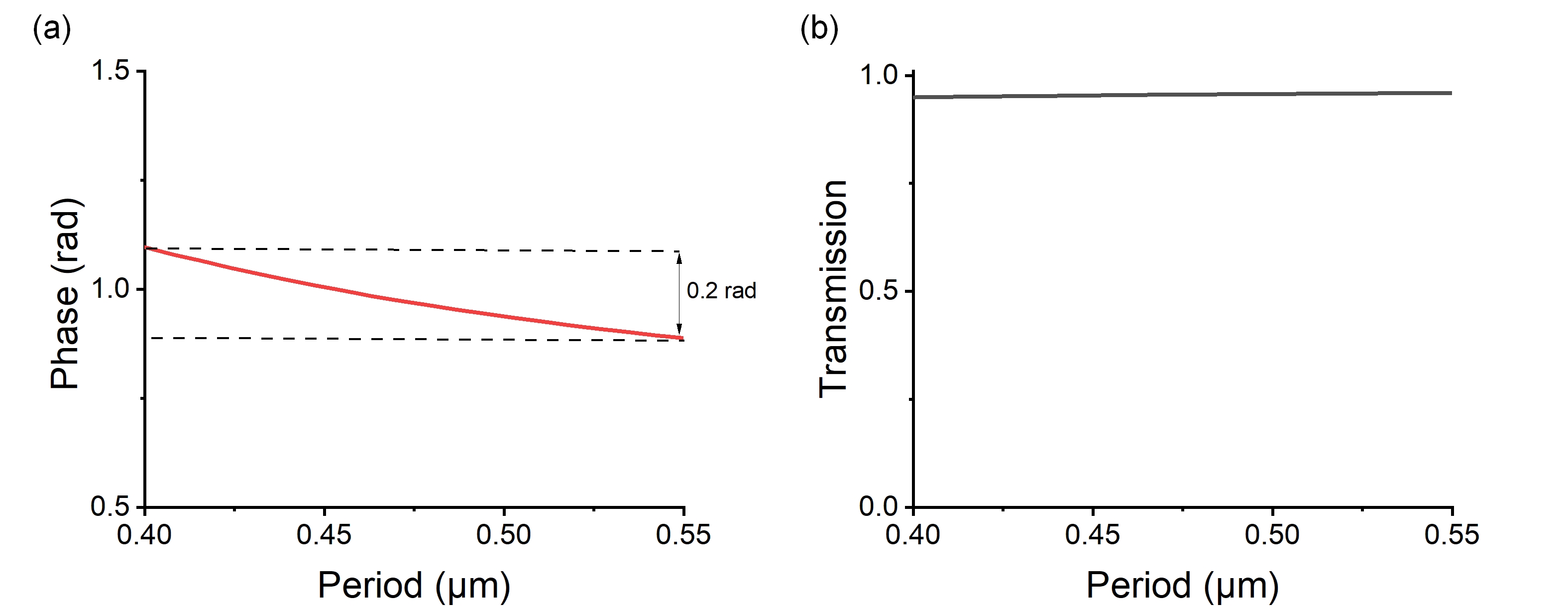
**

Supplementary Figure S2. The phase and transmission changes caused by stretching in the metasurface unit with a diameter of 0.12 microns. (a) The phase changes caused by stretching in the metasurface unit with a diameter of 0.12 microns. (b) The transmission changes caused by stretching in the metasurface unit with a diameter of 0.12 microns.

1. Uniform Stretching Device for Optical Performance Characterization and Imaging Characterization


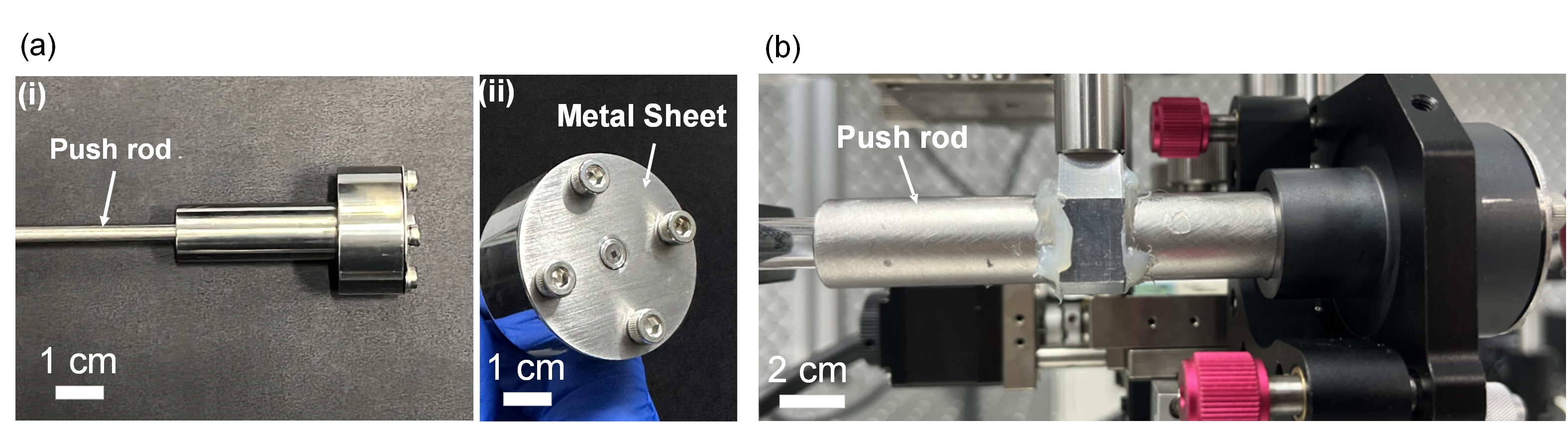


Supplementary Figure S3. Uniform Stretching Device for Optical Performance Characterization and Imaging Characterization. (a) Uniform Stretching Device for Optical Performance Characterization. (b) Uniform Stretching Device for Imaging Characterization.

1. Imaging results of the flexible metalens with 1× to 2× magnifications on the image at varying stretch ratios


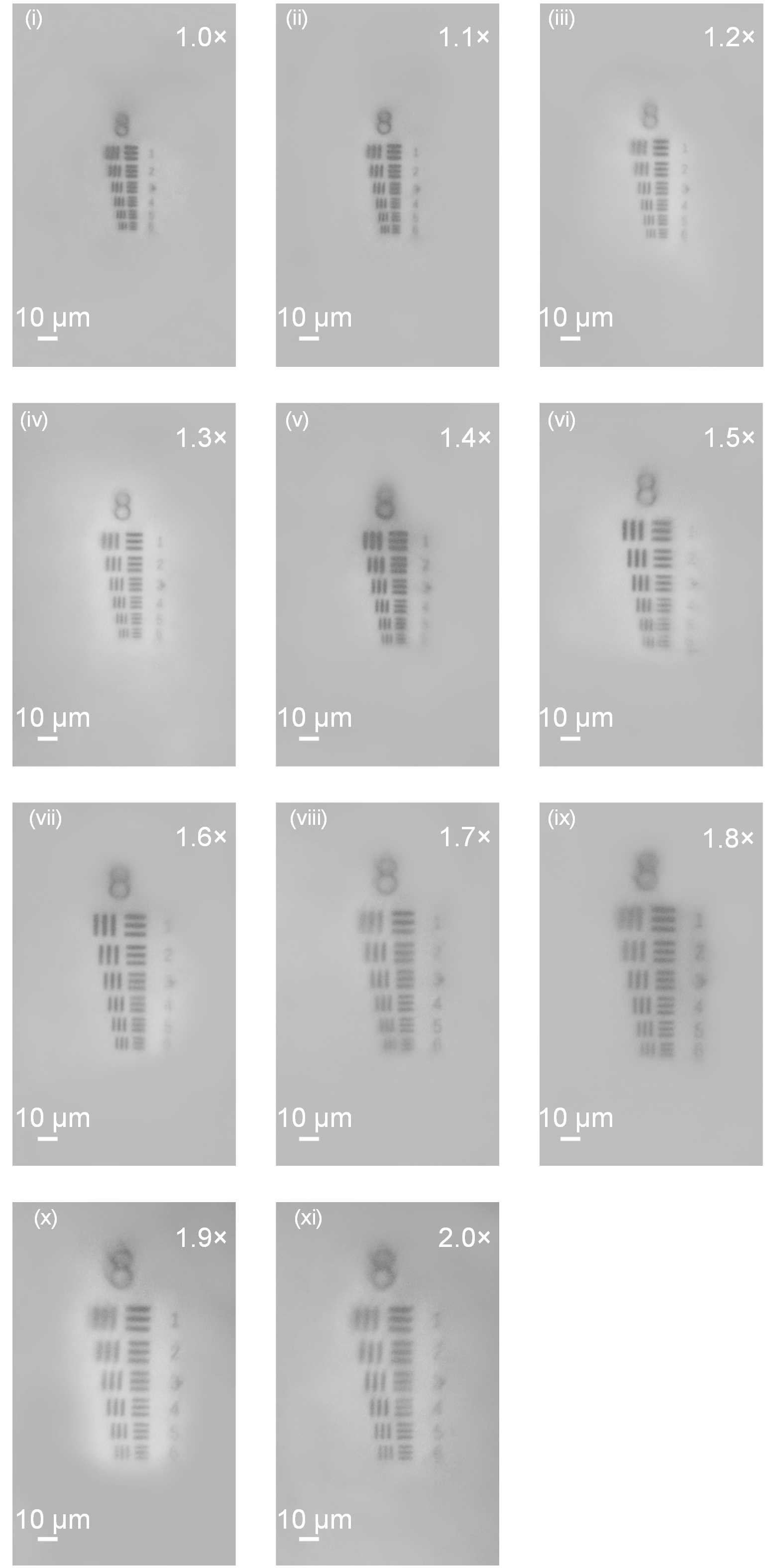


Supplementary Figure S4. The imaging results of the flexible metalens vary with the stretch ratios and exhibit magnifications ranging from 1× to 2×, as shown in (i) to (xi), respectively.

1. **The influence of the number of stretches on the performance of the flexible metalens**


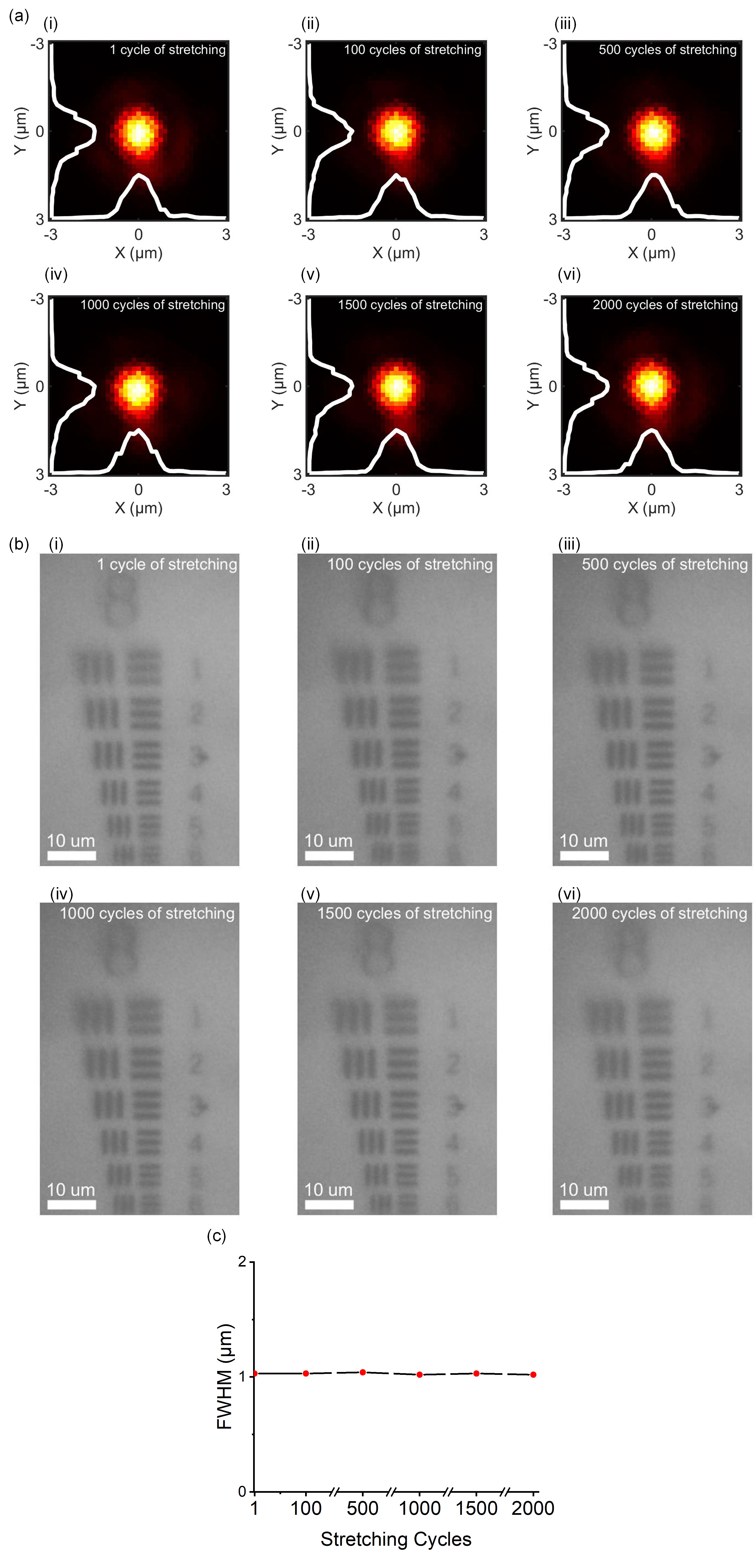


Supplementary Figure S5. Optical performance characterization of the flexible metalens after repetitive stretching tests. (a) Energy distribution of the flexible metalens at the focal plane after different cycles of stretching: 1 cycle, 100 cycles, 500 cycles, 1000 cycles, 1500 cycles and 2000 cycles as depicted in (i) to (vi). (b) Imaging results of the flexible metalens after different stretching cycles: 1 cycle, 100 cycles, 500 cycles, 1000 cycles, 1500 cycles and 2000 cycles as illustrated in (i) to (vi). (c) FWHM as a function of stretching cycles.
